# Risk-Based Quality Management: A Case for Centralized Monitoring

**DOI:** 10.1007/s43441-024-00719-1

**Published:** 2024-12-11

**Authors:** Nicole Stansbury, Danilo Branco, Cris McDavid, Jennifer Stewart, Kristin Surdam, Nycole Olson, Joanne Perry, Jeremy Liska, Linda Phillips, Amanda Coogan, Anina Adelfio, Lauren Garson

**Affiliations:** Association of Clinical Research Organizations (ACRO), 601 New Jersey Ave NW #350, Washington DC, 20001 USA

**Keywords:** Risk-based monitoring, Risk-based quality management, Centralized monitoring, Clinical trial quality, RBM, RBQM

## Abstract

Since 2019, the Association of Clinical Research Organizations has conducted a landscape survey of risk based quality management (RBQM) adoption in clinical trials. Here, we present data from four years of surveys, with an emphasis on the most recent: the 2022 survey included data from 4958 trials across seven contract research organizations, of which 1004 were new studies started in 2022. Results indicate that while overall risk assessment adoption is strong, it is lagging in other risk-based components which suggests companies are not deriving the full expected benefits of performing a risk assessment and mitigation process to their trials. The 2022 study also suggests new study starts showing promising traction, with adoption hovering near 50% for most RBQM elements. At the same time, the survey suggests industry has mixed views on the potential value of quality tolerance limits (QTLs). Ultimately, centralized monitoring is being underutilized despite the potential of increased patient safety oversight and improved data quality. The authors of this paper developed a case study based on a trial in clinicaltrials.gov to demonstrate how RBQM adoption could include the key RBQM elements such as centralized monitoring, reduced source data review and source data verification as well as implementation of QTLs in a real-world scenario. The authors believe the clinical trial industry has an obligation to utilize centralized monitoring to produce more efficient and effective clinical trials and will make a case to do so in this paper.

## Introduction

**Fig. 1:**
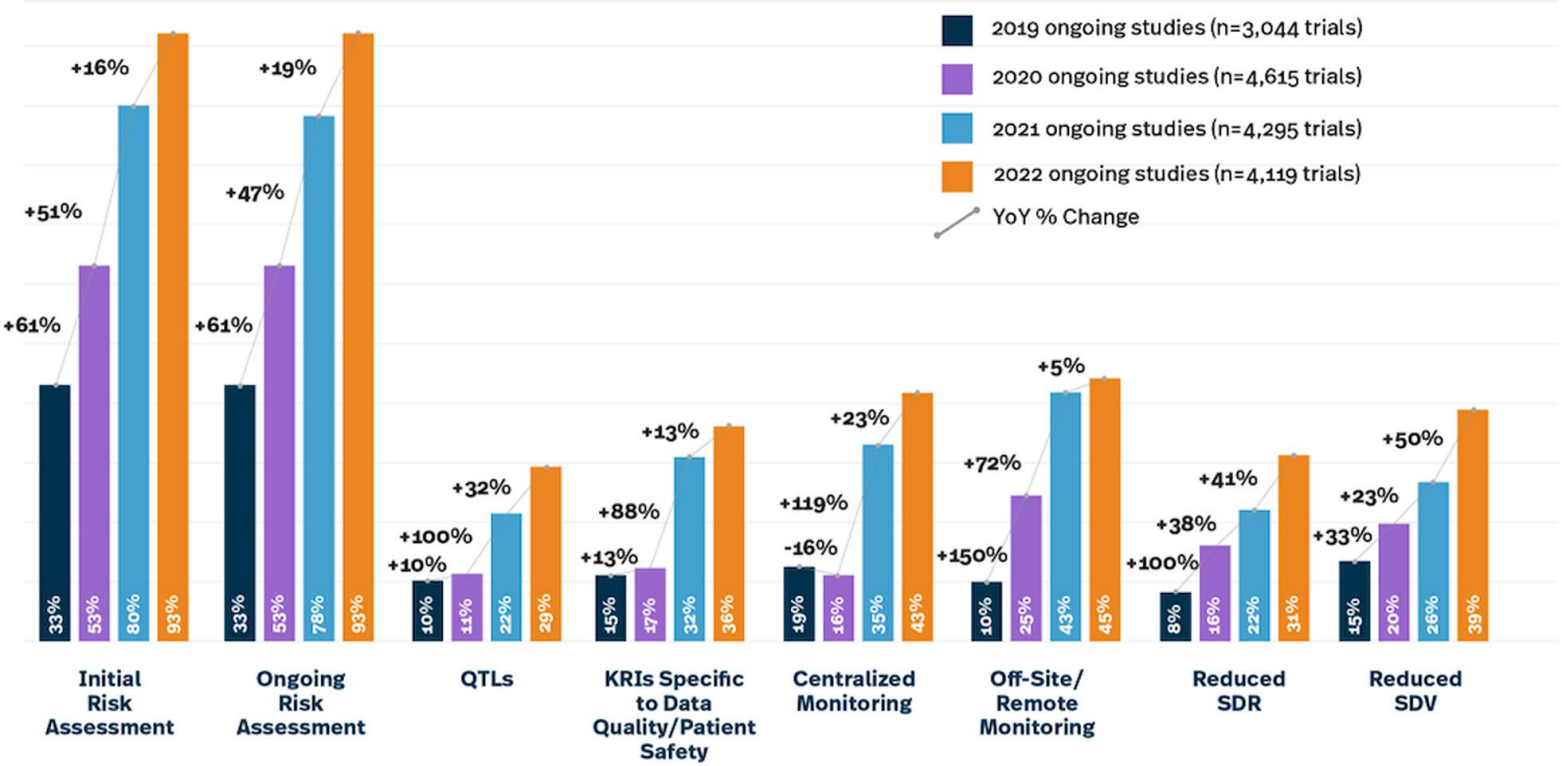
RBQM landscape survey results for ongoing studies 2019–2022

Risk based quality management (RBQM) in clinical trials centers on detecting, addressing, preventing, and mitigating risks and threats that could undermine patient safety, trial processes, and data integrity. The overall goal in this quality by design (QbD) approach is not to eliminate all errors, but instead to focus on reducing errors that matter and would otherwise threaten trial outcomes or patient safety. RBQM is a cross-functional framework enhanced by proactive risk monitoring and mitigation to ensure patient safety and overall data quality. Despite the fact that central monitoring can significantly help with overall data quality, adoption by industry is still sporadic and slow (Fig. 1).

The Association of Clinical Research Organizations (ACRO) is a trade association of global clinical research organizations (CROs) and technology companies. ACRO’s mission is to collaborate with regulators, policymakers, and other industry stakeholders, and help inform policy that fosters efficient, effective, and safe conduct of clinical research. We previously reported results in 2021’s Risk-Based Monitoring in Clinical Trials: Past, Present, and Future [1] and 2023’s *Risk-Based Monitoring in Clinical Trials: 2021 Update*, which includes all studies, all phases, and a wide range of indications and trial sizes. [2] The 2022 landscape survey has shown an overall, if uneven, rise in trials reporting at least one RBQM component. Our 2019 landscape survey of 6513 ongoing studies that year found just under half contained at least a single RBQM component; in our 2022 report on 4958 ongoing studies, more than three quarters contained at least a single RBQM component. The RBQM component definitions remained the same each survey year, in order to look at adoption trends over time. (See Table 1). Over the four years covered by our reports, an upward trend in all components was observed (See Fig. 1).

**Table 1:** Definitions [1]

Terminology	Definition
Centralized monitoring	The remote surveillance of aggregated electronic data, including data analysis, to determine if action is warranted. Common tools used in centralized monitoring are data visualizations that aid in identifying data anomalies and trends/patterns for further analysis
Reduced source data review (SDR)	Shift from 100% SDR to more focused monitoring where data captured in the source documents such as lab reports, medical records, etc., are reviewed to confirm compliance with the protocol and good clinical practices. Processes used by the site staff to collect data are also assessed
Reduced source data verification (SDV)	Shift from 100% SDV to more focused monitoring where data in the electronic data capture (EDC) system is confirmed to correlate directly to data captured in the source documents, such as lab reports, medical records, etc., by confirming the accuracy of the data transcription from source to EDC

**Fig. 2:**
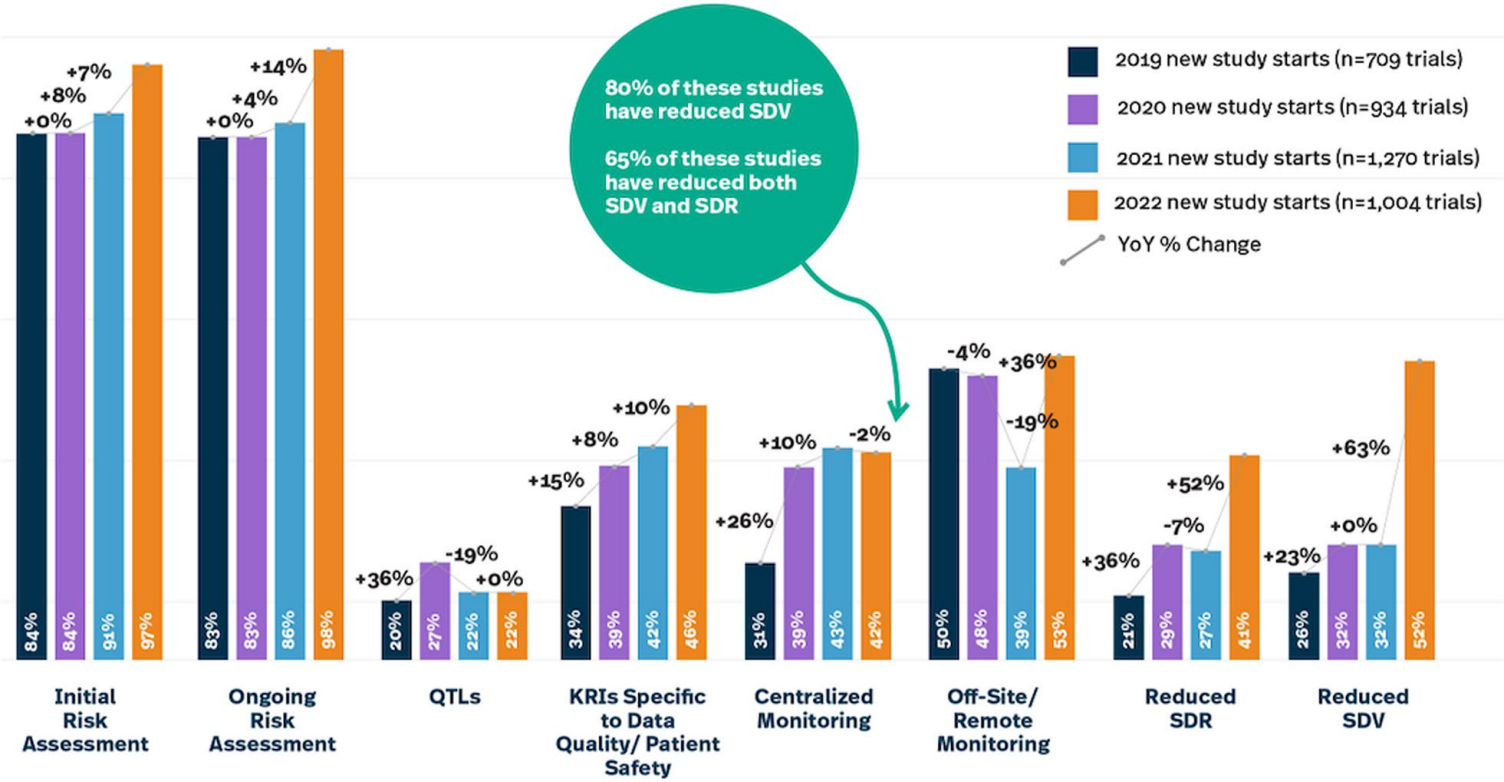
RBQM landscape survey results for new study starts 2019–2022

There are some important call outs to note in the data. The survey in 2022 found 65% of new study starts which have adopted centralized monitoring also reported reducing both SDR and SDV (Fig. 2). Interestingly, between 8 and 18% of new study starts reduced SDR and/or SDV without implementing centralized monitoring. This was common practice beginning around 2005 when risk-based monitoring was first gaining traction and companies were sampling subjects, subject visits or data points and the data visualization tools and technologies which support centralized monitoring were unavailable, immature and/or unproven in their value and return on investment. However, centralized monitoring was introduced in FDA’s 2013 guidance, *Oversight of clinical investigations—a risk-based approach to monitoring* to supplement and/or reduce the extent or frequency of onsite monitoring. The industry best practice among CROs and many sponsors has been to supplement any reductions in SDR and SDV with centralized monitoring to increase confidence in patient safety oversight and data integrity.

To quote the guidance, “a recent review of on-site monitoring findings collected during a multi-center international trial also suggests that centralized monitoring can identify the great majority of on-site monitoring findings. The review determined that centralized monitoring activities could have identified more than 90% of the findings identified during on-site monitoring visits” [3].

In early implementation of reductions in SDR and SDV, it was common for SDV to be reduced slightly (i.e., 70% of data undergoing transcription checks) but SDR to still occur at or near 100%. As centralized monitoring processes, tools and technologies have become more sophisticated and efficient in their ability to aggregate data across data sources and improve identification of issues and to monitor for data outliers, anomalies and trends, the reductions in SDR and SDV have continued to increase. Today, it is common to see sampling of patients or patient visits for SDR and not uncommon for some patients or patient visits to undergo very little or no SDV. It is virtually undisputed that centralized monitoring provides capabilities that are more timely, accurate, reliable, valuable and efficient than traditional onsite monitoring activities like SDR and SDV. It is important to note that onsite monitoring, including sampling of SDR/SDV is still recommended in most studies, but we continue to see steady reductions.

## A Call to Action

The time is now for CROs, sponsors, sites, and regulatory agencies to work together more collaboratively to increase adoption of RBQM. Study complexity is increasing across all phases in the number of endpoints collected, procedures performed, and patient visits required [4]. The return on investments made in the development of these treatment options is decreasing due to the additional time and budget required to complete trials. Data volume and increasing data sources are prohibitive to manual practices utilized by clinical research associates (CRAs) to monitor for patient safety and data integrity. To sustain the work required to deliver life changing and life-saving treatment options to patients, we must improve the efficiency of our work. The clinical trial industry must come together to educate each other on the value of RBQM. Sharing case studies, success stories, and other insights gleaned from real world activities can be an effective way to advance comfort with and adoption of RBQM to the benefit of patients and the clinical trial industry.

The ACRO RBQM Working Group has developed a case study designed to illustrate some of the many benefits of leveraging these tools and best practices. We selected a representative trial from clinicaltrials.gov [5] and developed a real-world RBQM strategy for implementation.

The clinical trial we selected from clinicaltrials.gov included a protocol synopsis, a description of primary safety and efficacy endpoints, key eligibility criteria, and a description of visits and procedures. We simplified the study design and eligibility criteria for the purposes of this case study and made some assumptions on the schedule of events and how data would be collected. We wanted to demonstrate how this works in cases where data is captured in multiple electronic sources.

The purpose of the clinical trial used in this case study was to evaluate two different antibiotics, for the outpatient management of uncomplicated skin and soft tissue infections (uSSTIs or SSTIs) in children and adults.

The ACRO RBQM Working Group is comprised of representatives from clinical operations, data management, centralized monitoring, quality assurance and technology vendors. Our team worked together on the case study to:

- Determine the critical data and process
- Identify risks to the critical data and critical process
- Suggest the most effective and efficient ways to mitigate risks to critical data and critical process, specifically focusing on centralized monitoring as a key mitigation strategy
- Develop a SDR/SDV sampling plan relying on centralized monitoring to supplement reductions in SDR and SDV

We included a process to triage data or safety concerns discovered by central monitors or CRAs to data management, medical monitors and biostatistics for input and further investigation if needed.

## Identifying Critical Data and Critical Process

We defined critical data as the data used to collect or support the primary and secondary study endpoints and support inclusion/exclusion criteria (Table 2). Critical process was determined to be the processes associated with the collection of the endpoint data as well as processes supporting informed consent, confirmation of eligibility and processes associated with the dispensation, administration and collection of investigational product. Also considered critical are the processes used to collect, assess, document and report adverse events and concomitant medications. Once critical data and critical processes were identified, we worked to assess risks to the critical data and critical process.

**Table 2:** Identified critical data and critical processes

Visit	Critical data	Critical process
Visit 1– Screening/baseline	Data supporting inclusion and exclusion Criteria: medical history, physical exam, concomitant medications, labs (WBCs), vitals (body temperature) SSTI symptom assessment by the patient: tenderness, pruritis, erythema, purulent draining, swelling, local warmth Physician measurements	Informed consent Verification of eligibility Confirmation of diagnosis (abscess or cellulitis) Education of patient/caregiver on diary completion/assessments IP dispensation/randomization
Visit 2– Wound check (virtual check in)	Patient electronic diary collection: daily IP dosing, vitals Daily SSTI symptom assessment AEs (including fevers or new/worsening of skin infections) Concomitant medications	Completion/assessments Education of patient/caregiver on diary AE evaluation Decision for additional intervention/withdraw
Visit 3– End of therapy	Vitals (body temperature) SSTI symptom assessment by the patient: tenderness, pruritis, erythema, purulent draining, swelling, local warmth Physician’s global assessment and measurements AE and concomitant medication collection	IP collection AE evaluation Physician’s global assessment and measurements
Visit 4– Test of cure/early termination	Vitals (body temperature) SSTI symptom assessment by the patient: tenderness, pruritis, erythema, purulent draining, swelling, local warmth Physician’s assessment of cure AE and concomitant medication collection	Physician’s global assessment and measurements Physician’s assessment of cure
Visit 5– End of study	AE and concomitant medication collection Labs (if needed) Physician’s assessment of cure	AE evaluation Physician’s assessment of cure

## Identifying Risks to Critical Data and Critical Process

Here are some examples of the risks that were identified to critical data and critical process:

- Due to the varying age ranges allowed in the trial, there could be several different consent versions required: a caregiver consent, an assent for minors and a patient consent. There is a risk of the site not utilizing the appropriate consent version which could impact the patient’s rights and the ability to use the data captured for analysis.
- The specific type of SSTI (cellulitis or abscess) and the size of the skin infection determines the randomization stratification for the trial. There is a risk that the physician and site staff could mis-randomize a patient if care and attention is not appropriately applied during the baseline visit.
- Physician’s Global Assessment and Assessment of Cure should be performed by the same physician for a patient to ensure consistency in how scoring is conducted. If different physicians complete the assessments for a patient, results may not be reliable, impacting the integrity of the trial.
- Patient’s adherence to the IP dosing schedule is critical to ensure a therapeutic dosage and prevent worsening of the skin infection. There is a risk that the patient does not comply with the dosing schedule which could impact the trial results or create a safety issue for the patients. Additionally, due to the varied randomization schema and dosing regimen, site staff could provide patients with incorrect or confusing dosing instructions which could impact the trial results or patient safety.
- Patient’s Symptom Assessment (particularly within the first 48 h) is critical to monitor for worsening of the skin infection, additional infections, additional skin infection interventions or IP related Adverse Events (AEs). If worsening or additional skin infections are identified, patients should be assessed and potentially withdrawn from the trial to receive additional intervention. There is a risk that patients fail to comply with assessment requirements and/or site communications during the critical time period and this could impact patient safety or trial integrity.

## Mitigating Risks

We considered the following as we determined the most effective and efficient mitigations (including leveraging modern technologies) and attempted to establish some guiding principles to prevent duplication across functions as needed:

- Volume of data (higher volume = more automated methods such as use of electronic technology or programming of edits)
- Availability of data (if data was available centrally, then it should be reviewed centrally)
- Timing of data availability (review centrally if available instead of waiting for onsite reviews due to the short duration of patient participation and the critical evaluation for worsening and new AEs within first 48 h of dosing)
- Importance of correlating certain data across different sources (eDiary data, central lab data and Physician Global Assessment from EDC)
- Resources best positioned for “real time review.” Consider cost implications by reserving expensive, specialized resources for performing mitigations that have been triaged or that can only be reviewed by staff with a specialized skillset.

After completing the risk assessment, risk mitigation strategies generally fell into six categories (Table 3).

**Table 3:** Risk mitigation strategies

	Protocol improvements	• Include the use of eConsent to help manage the multiple consent versions needed due to the age range of the study population • Include e-Diary for patients to capture patient assessment, monitor body temperature, collect AEs and to assess IP administration compliance • Include Integrated Randomization Technology (IRT) to help manage randomization stratification
	Site & CRA training	• Train site staff to train patients on the importance of monitoring for symptom worsening and dosing compliance • Train CRAs and site staff on the importance of the same physician rating within each patient • Train Investigators on the importance of the initial diagnosis (cellulitis vs abscess)
	Data management plan	• Ensure eDiary set up for patient dosing, AE and symptom assessments meets details needed for the study • Ensure reconciliation between eDiary and EDC for management of AEs • Ensure reconciliation of lab data, particularly for culture results • Assess for study level trends in missing data • Program edits to identify discrepancies with physician assessment and test of cure
	Centralized monitoring plan	• Utilize patient profiles to confirm protocol eligibility criteria to the extent possible across eConsent, IRT, Medical History, Concomitant Medications, Physical Exam (include skim assessment and measurements), vitals and lab results • Utilize patient profiles and data visualizations to identify protocol deviations for triage to CRA for site staff (or patient) retraining • Monitor for concerns with medical care of patients, particularly those related to study withdraw criteria, for triage to medical monitor • Monitor patient diary data daily for compliance, signs and symptoms of worsening of skin infection, dose interruptions or other non-compliances for escalation to site staff for immediate follow up and/or triage to medical monitor • Monitor physician skin assessments for inconsistencies in assessments over time which may indicate different assessors or transcription errors • Monitor visualizations comparing physician assessment to patient assessment for extreme lack of correlation • Assess sites for key risk indicator (KRI) trends in missing data, rate of drug interruption, rate of patient discontinuation • Assess study for Quality Tolerance Limits (QTLs) such as %/# patients discontinuing prior to Visit 3
	Medical management plan	• Assess for trends in AEs related to IP • Assess for trends in SAEs and resulting in discontinuation • Discuss escalations from CRAs/central monitors related to eligibility, medical care of subjects, withdraw criteria
	On-site monitoring plan (including SDR/SDV and site management)	• Confirm site processes for ICF and source documentation for confirmation of eligibility criteria, including good source documentation practices (ALCOA-C) • Confirm sites process for collecting primary and secondary endpoints including ensuring consistent physician performs skin assessment for a patient • Confirm appropriate experience, training, oversight and delegation of site staff • Confirm IP storage, dispensation, administration and collection • Confirm regulatory documentation and reporting compliance • Sample SDR and SDV for accurate and complete transcription to EDC • Discuss non-compliances, safety concerns, query and action items with site staff for resolution

## A Focus on Centralized Monitoring Mitigations

Our goal was to create a robust, cross-functional RBQM strategy that leveraged the benefits of centralized monitoring to reduce the extent or frequency of onsite monitoring. With only 5 visits per patient over a 6-week period, we needed a way to closely monitor patients for safety and efficacy compliance that is an improvement over traditional monitoring without requiring frequent onsite visits. We created some sample data and data visualizations to highlight the benefits of a centralized monitoring approach. We were able to do this by designing and demonstrating a strategy that relies on aggregating data from multiple sources into visualizations that allow central monitors to identify issues more easily than through traditional monitoring [6].

**Fig. 3:**
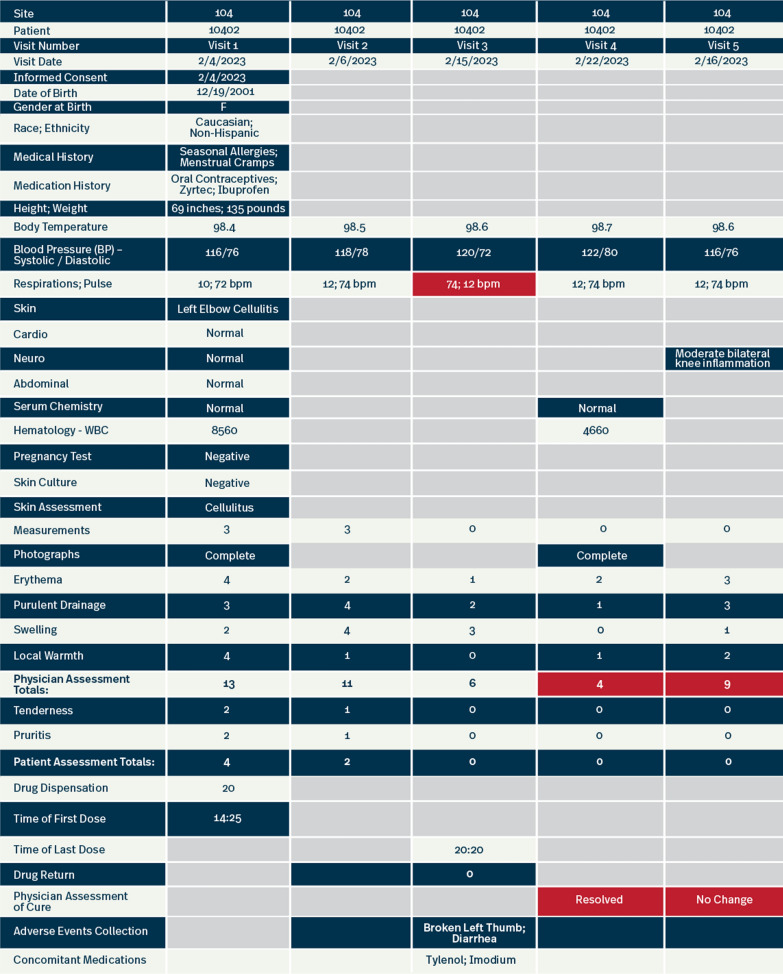
Example of a patient profile

In the sample visualization (Fig. 3), which represents a patient profile, you can see each column represents data collected for a specific visit for the patient. In addition, the rows display data coming from several different sources: electronic data capture (EDC) data, central lab data and eDiary data. The format of a patient profile is such that central monitors can look at data for a patient within a visit and across visits to identify non-compliances such as eligibility concerns, prohibited meds, and missed procedures, as well as to associate medical history and AEs with concomitant medication (and vice versa). In this example, the visualization has been programmed to highlight discrepancies (see cells highlighted red in Fig. 3) in vital signs that could be indicative of a transcription error or a potential AE. Patient profiles such as this can be useful in helping review site responses to data management queries and providing an overall view of the “big” picture of the journey of the patient throughout their participation on the trial. This type of view of a patient’s data is not possible during traditional monitoring performed by CRAs onsite using multiple source documents and form by form reviews in EDC that focus on a single visit or procedure at a time.

**Fig. 4:**
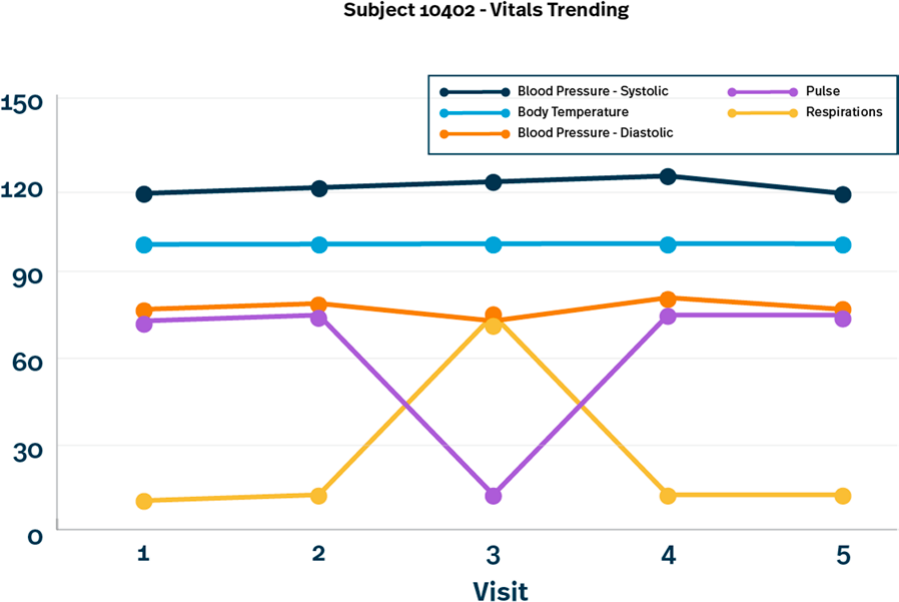
Vitals trending example

Other ways to view patient data through centralized monitoring are to graphically view data to assess for trends. In the example (Fig. 4), we have taken the vital signs for the same data displayed in the patient profile (Fig. 3) to show other ways data can be displayed to identify unexpected data trends which would prompt investigation.

While data management queries are often used to programmatically identify errors in data such as the examples here, often site staff will respond with “ok as is” simply because they do not recognize the subtleties of simple transcription errors, especially if these errors were misrepresented in the source itself. By looking at the data graphically, the errors become more obvious. This is a very simple example, but next, let’s look at a more complex example.

**Fig. 5:**
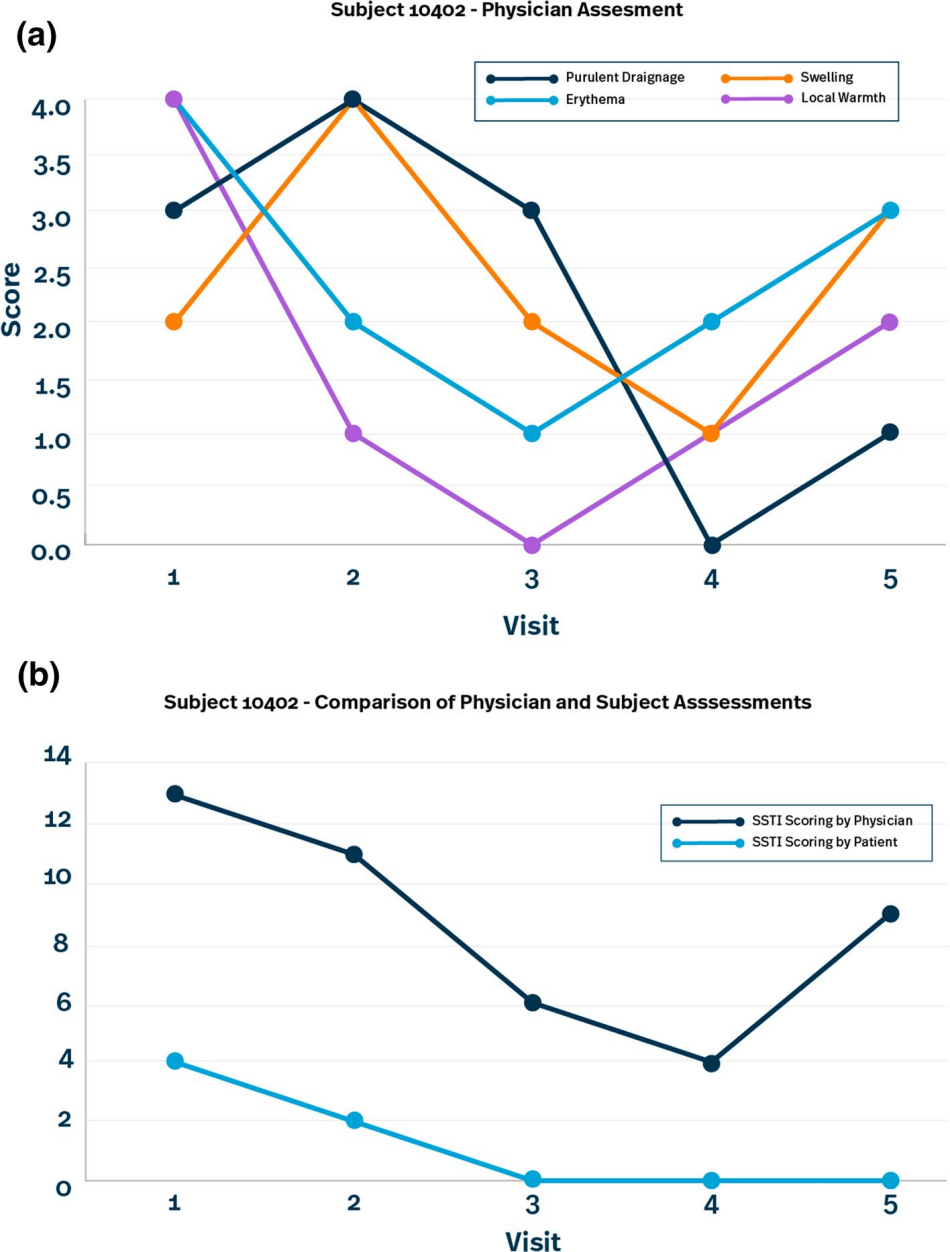
**a** Physician assessment example. **b** Comparison of physician and subject assessments example

In the example below (Fig. 5a and b), we are trending physician skin assessments over time for a specific patient. Neither CRAs in the field, nor data management via programmed edits, could pick up on inconsistencies in the scoring over time. With centralized monitoring visualizations, it can become apparent that the physician ratings are not as expected. Typically, we would expect to see consistency in either improvements or worsening across each symptom being assessed over time. We would also expect these to correlate with both the test of cure assessment (resolved, improved, no change or worsened) and/or the patient’s symptom assessment. In this example, the assessments fluctuate from visit to visit with some worsening and some improving. In addition, the final assessment does not correlate with the patient symptom assessment over time, nor does it correlate with the test of cure assessment at visits 4 and 5 (resolved and no change respectively) or the measurements of diameter collected over time (1 and 2 mm respectively). This complexity may be too difficult to programmatically detect, but using centralized monitoring visualization, this can initiate further discussion with the site to better understand the data. After investigation, we may discover a transcription error (e.g., the wrong patient’s data reported in EDC for a visit or two), changes in physician raters within the patient at the site, or confirm that this is actually what is documented by the site to have occurred.

In addition to the benefits of aggregating data to look at patient profiles and trending data within a patient, centralized monitoring visualizations can help identify sites that are outliers in both clinical and operational data. Centralized monitoring can accomplish this by utilizing Key Risk Indicators (KRIs) and to identify data concerns that could impact overall study data integrity or patient safety by creating visuals to monitor for QTLs. Here are some examples of the potential KRIs and QTLs the ACRO RBQM Working Group proposed for this sample case study (See Table 4).

**Table 4:** KRIs and QTLs

Routine KRIs	Possible QTLs (with acceptable ranges): *Recommendation is to pick 2–3 QTLs that focus on endpoint integrity, patient safety, enrolling the right patients*
• AE/SAE/AEs of special interest Rates • Early Termination rate (including termination due to AEs) • Protocol deviation (PD) rate • Important PD rate • Data change rate • Query rate • Rate of missing endpoint data • ‘SDV backlog’ or ‘participants visits since last monitoring visit (MV)’. This is a volume-based risk indicator that helps to drive for-cause or anticipated onsite monitoring	• % randomized patients with IP related AE/SAE • % of non-correlated SSTI / PGA scores • % of missing/invalid skin cultures • % of patients terminated from the trial due to related AEs • % of patients lost to follow up (LTFU) • % of patients mis-randomized or mis-stratified • % of patients with endpoint errors/deviations • % of patients with less than 85% IP compliance • % of patients enrolled with eligibility violations • % of patients with dose reductions or interruptions, due to an AE or SAE

## Connecting Centralized Monitoring to Reductions in SDR and SDV

By developing a robust centralized monitoring strategy, we can more confidently implement a reduced SDR/SDV sampling strategy for CRAs to follow in their onsite or remote monitoring of patient data. This makes monitoring large volumes of data more manageable as CRAs focus on critical data and processes. In addition, many sites are placing limitations on the frequency, duration and number of CRAs that can be present onsite or the number of hours or days that a CRA can have access to electronic health records (EHRs) for remote monitoring. A robust approach of centralized monitoring and SDR/SDV sampling is the solution to the pressures caused by increased data volume and limited access to sites. Sampling strategies can be data point sampling, procedure sampling, subject visit sampling and/or subject sampling.

A good sampling strategy ensures that CRAs spend less time onsite performing transcription checks and more time performing comprehensive review of entire subject visits to focus on critical processes such as outlined in the Table 2 above.

**Fig. 6:**
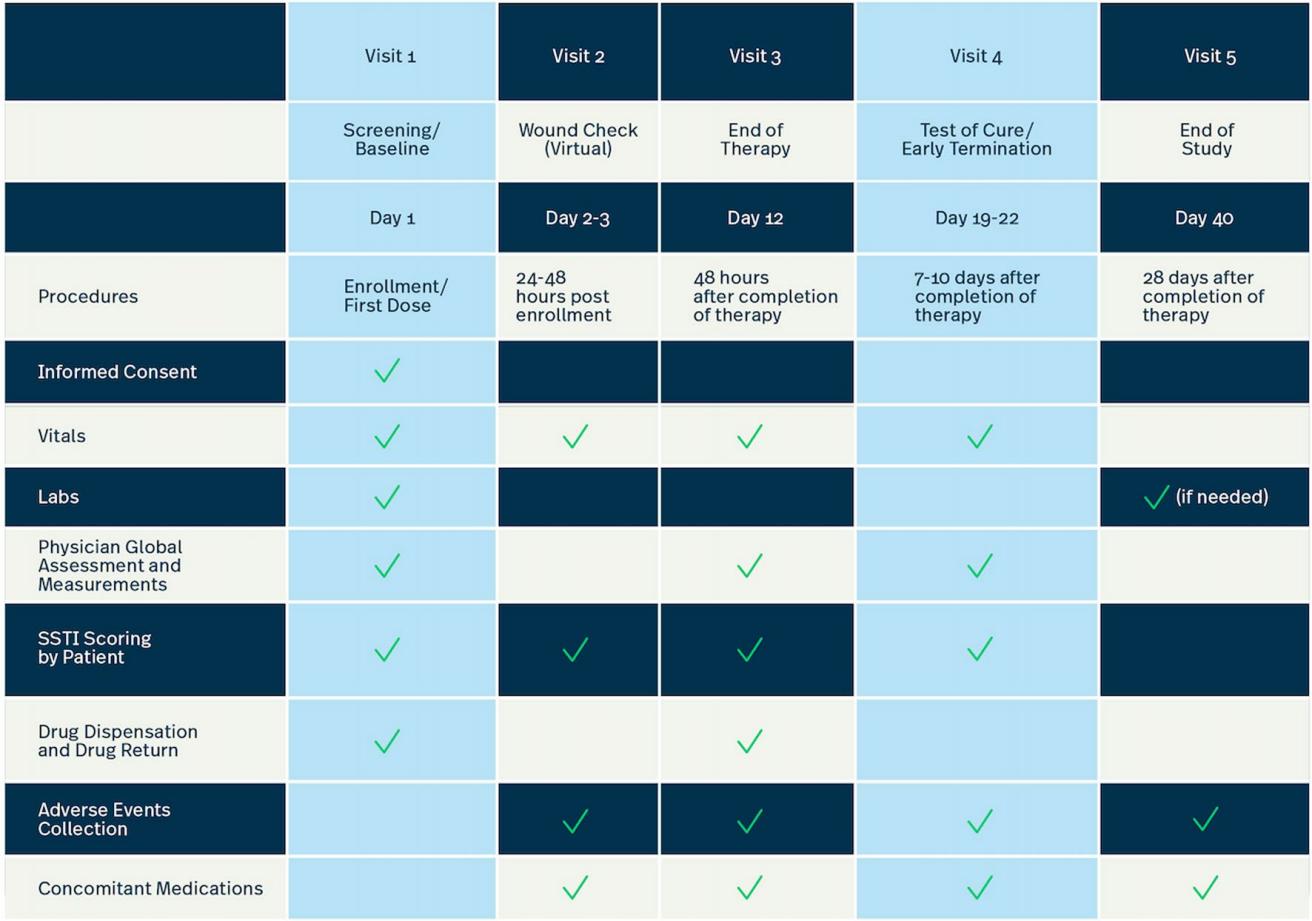
Schedule of events with visits 1 and 4 selected for SDR/SDV patient visit sampling strategy. This schedule of events denotes which procedures should occur on which visit. It is not an exhaustive list of procedures for each visit, but instead it is meant to demonstrate some examples. Highlighted columns (visits 1 and 4) were selected for SDR/SDV sampling

The following table (Fig. 6) demonstrates the subject visit sampling approach we would recommend for our case study. Based on the risks we identified and our mitigations through centralized monitoring and the collection of data via eConsent, eDiary and Integrated Randomization Technology (IRT), we recommend that CRAs still perform 100% SDR and SDV of Visits 1 and 4 for all patients. Visits 2, 3 and 5 can be monitored centrally by central monitors looking at the data “in near real time” to alert CRAs to site and patient issues requiring their attention.

Visit 1 ensures CRAs will cover the assessment of site compliance with good clinical practices, protocol and good documentation practices. They can confirm consent, eligibility, IP management and dispensation and evaluate the physician’s assessment documentation, accuracy of transcription to EDC and patient training. Visit 4 is the test of cure visit which serves as the primary endpoint.

During the conduct of their monitoring reviews, central monitors may create queries for sites and/or action items for CRAs to investigate any data anomalies that were discovered. Should significant concerns be identified, including a large volume of action items or queries for critical data for a site, an onsite interim monitoring visit (IMV) could be prompted to expand SDR and SDV to specific data within visits 2, 3 or 5 for a patient(s).

In addition to CRAs performing SDR/SDV sampling which include focus on critical process, in our model, they will also investigate targeted findings created by central monitors. This ensures a comprehensive approach to protecting subject rights, welfare and safety and ensuring the integrity of the trial data. While no monitoring method guarantees perfection, regulatory authorities expect monitoring strategies to identify and remediate errors that matter.

## Triage to Medical Monitors and Biostatistics

These specialized team members help us to evaluate the impact that errors and omissions might have on patient safety and the integrity of data on the trial. The following are examples of how that triage process and assessment works in an efficient and effective RBQM strategy.

- Central Monitors and CRAs will escalate any patient safety concerns to the medical monitor for additional evaluations. Medical monitors may use additional data visualizations such as patient profiles to review concerns related to an individual patient or study-level visualizations to assess trends in adverse event (AE)/serious adverse event (SAE) reporting, AE type, relationship or timing correlated to dosing or discontinuation.
- Data management staff will escalate any trends in missing endpoint data to the biostatistician for evaluation on the impact to trial integrity. This includes monitoring of excursions to any thresholds set for quality tolerance limits.

In this strategy, by starting with critical data and critical process, then developing fit for purpose mitigation strategies, we have created a cost-effective monitoring solution that identifies and remediates errors that matter.

## Increasing Patient Safety and Data Quality while Achieving Cost Efficiencies

This robust RBQM strategy has the potential to reach the desired “trifecta” in an innovative clinical trial model as compared to traditional monitoring models. By first identifying the critical data and process, then assessing the risks to that critical data and process and mitigating/monitoring those risks using a combination of centralized monitoring and reduced SDR/SDV, we can hopefully create operational efficiencies that improve oversight of patient rights, welfare and safety and better ensure data integrity.

Oversight of patient rights, safety and welfare increases with centralized monitoring with the aggregation of data across a number of different data sources such as EDC, central labs, ePROs/eDiaries that can best be accomplished through centralized monitoring. In addition, centralized monitoring tools and visuals can compare data across those different data sources, highlighting data that represents a trend within a patient or across patients for further investigation. Centralized monitoring can occur within days of a patient visit if sites comply with data entry timelines. This has dual benefits of driving sites to improve data entry timeliness, but also allows for rapid identification and escalation of any safety issues which could sit unnoticed for weeks or months until a CRA conducts an onsite or remote monitoring visit.

Reducing SDR and SDV to focus on a sampling of patients or patient visits allows CRAs to spend valuable and limited time during onsite or remote IMVs reviewing and evaluating that each site has consistently followed a process for:

- Administering informed consent
- Verifying and documenting eligibility requirements have been met
- Collecting and reporting AEs and SAEs

Centralized monitoring allows for better oversight of data integrity by presenting data in a visual format that highlights unexpected or lack of variation in data over time. Data visualizations can even help to identify potential transcription errors and better show the overall impact of data anomalies or missing data across the study. Prompt review of data allows for the identification of protocol deviations to be discovered, escalated to sites and addressed before those deviations are repeated across other patients which could impact the trial outcomes.

Reducing SDR and SDV using a sampling approach can improve data integrity by allowing CRAs to focus on data and data associations within a patient visit as they look for documentation on how a site performs critical procedures, who performs those procedures, how they are documented and if a valid process was used to collect the data captured in the EDC system or other source systems. This monitoring methodology emphasizes SDR (process and documentation) over SDV (transcription).

Using centralized monitoring and reduced SDR/SDV has the potential to further provide operational efficiency benefits:

- Centralized monitoring allows a large portion of the trial monitoring to occur on an ongoing and continuous basis avoiding large backlogs of data and data issues.
- Centralized monitoring helps sponsors in demonstrating continuity in oversight of patient safety and data integrity.
- SDR and SDV sampling helps to ensure that when a critical process issue, patient safety concern or other non-compliance is identified during a monitoring visit, a CRA will have time to work with the site to bring them back into compliance without significantly impacting their data review backlog.
- Efficiencies can be amplified by combining a sampling strategy with sites who can provide remote access to source documents via direct access to EHR and/or eSource without any additional burden.
- Centralized monitoring can be deployed in cost effective regions using highly experienced and trained team members which has the possibility of helping to control clinical monitoring costs

## Conclusion

The purpose of this case study is to demonstrate the significant benefits of centralized monitoring in combination with a reduction in SDR and SDV in clinical trials. This approach highlights potential operational efficiencies that can enhance the ability to monitor patient safety and ensure data integrity. The authors believe it is not only an imperative to utilize centralized monitoring, but an obligation to patients and trial integrity.

The time is now to more fully embrace RBQM. The ACRO RBQM Working Group member companies have individually found RBQM strategies to be effective and offer advantages over more traditional monitoring methods, while continuing to ensure patient safety and data integrity. Later in 2024, we will be publishing our most recent landscape data report; a preliminary look suggests current trends are continuing in terms of rising adoption rates and subsequent operational benefits.

By sharing best practices and lessons learned we hope to accelerate the adoption of new ways of working which will ultimately have a beneficial impact on all clinical trial patients and others, who are later prescribed a vetted drug, device or biologic approved for use by regulatory authorities.

## Data Availability

ACRO owns and holds the landscape data that is provided in this manuscript. Additional data can be found at www.acrohealth.org or is available upon request.
